# Involvement of Circulating Cell-Free Mitochondrial DNA and Proinflammatory Cytokines in Pathogenesis of Chronic Obstructive Pulmonary Disease and Lung Cancer

**DOI:** 10.31557/APJCP.2021.22.6.1927

**Published:** 2021-06

**Authors:** Olga Bulgakova, Asemgul Kausbekova, Assiya Kussainova, Nurtas Kalibekov, Dulat Serikbaiuly, Rakhmetkazhi Bersimbaev

**Affiliations:** 1 *L.N.Gumilyov Eurasian National University Institute of Cell Biology and Biotechnology, Nur-Sultan, Kazakhstan. *; 2 *National Research Oncology Center, Nur-Sultan, Kazakhstan. *

**Keywords:** Circulating cell, free mitochondrial DNA, Chronic obstructive pulmonary disease, lung cancer, interleukin-6

## Abstract

**Objective:**

Circulating cell-free mitochondrial DNA (cf-MtDNA) has been reported in patients with chronic obstructive pulmonary disease (COPD) and lung cancers. However, inter-relationships among the three biological events have not been well-characterized. Therefore, our investigation was conducted to better understand the role of cf-MtDNA on pathogenesis of the two diseases.

**Methods:**

Plasma samples were collected from 64 non-small cell lung cancer (NSCLC) patients (before therapy), 45 patients with COPD and 62 healthy individuals. cf-MtDNA copy numbers were detected using quantitative real-time polymerase chain reaction (qRT-PCR) and cytokines were determined using a human ELISA kit.

**Results:**

Our data indicate that smoking statuses of the patients and controls were significantly associated with increased cf-MtDNA in plasma samples. Furthermore, NSCLC patients had significantly higher cf-MtDNA copy numbers than COPD patients (p < 0.03) and normal controls (p < 0.02), together with elevated proinflammatory cytokines over the controls (p < 0.05). Our study shows that the copy numbers for the NSCLC patients were positively associated with their subsequent metastasis but inversely associated with their overall survival.

**Conclusion:**

Our study indicates certain lung injury (e.g., from cigarette smoking) was responsible for the release of cf-MtDNA and proinflammatory cytokines into plasmas among our patients and controls. The increase in cf-MtDNA copy numbers was significantly associated with the development of both COPD and NSCLC, with increase in interleukin 6, and from our 5-year follow-up, with poor prognosis among the NSCLC patients. Therefore, with further validation, cf-MtDNA can be considered for use as diagnostic and prognostic biomarkers for NSCLC.

## Introduction

Chronic obstructive pulmonary disease (COPD) and lung cancer are two of the major chronic diseases in many countries around the world, including Kazakhstan (World Health Organization, 2020) [https://www.who.int/news/item/09-12-2020-who-reveals-leading-causes-of-death-and-disability-worldwide-2000-2019]. In addition, cigarette smoking is well-recognized to be a major contributing factor to both diseases, especially for non-small cell lung cancer (NSCLC). Therefore, COPD and NSCLC are somehow etiologically related (Parris et al., 2019). In fact, COPD has been reported to be an independent risk factor for NSCLC, therefore there are NSCLC cases without any history of COPD (Tang et al., 2020). Consequently, such scenario has created opportunities for investigations on mechanistic relationships between COPD and NSCLC, especially on identifying new biomarkers which can be used to identify the diseases and to provide prognostic values. Our investigation was focused onto characterizing the usefulness of circulating cell-free mitochondrial DNA (cf-MtDNA) and cytokines for COPD and NSCLC. 

It is well-known that cigarette smoke contains many mutagenic and toxic chemicals which can cause DNA damage, cell death and abnormal biological functions. Indeed, these abnormalities would induce various types of damages, e.g., lung barrier dysfunction and tissue remodeling, as possible mechanistic linkages between smoking and COPD as well as NSCLC (Hou et al., 2019). 

Induction of cell death and/or tissue damage by smoking or by other mechanisms can also cause damage to mitochondria, leading to leakage of cf-MtDNA into body fluid, such as blood and urine. Indeed, the presence of cf-MtDNA in body fluids have been reported for a variety of chronic diseases, including COPD and lung cancer (Thurairajah et all., 2018; Jiang et al., 2020; Riou et al., 2020; Zhang et al., 2020). However, contradictory findings have been reported. For example, cf-MtDNA copy numbers in blood plasma were lower in patients with lung cancer than that in controls (Chen et al., 2018).

Another component which contributes to the scenario is the involvement of inflammatory factors, such as cytokines. Recently, it was shown, that NKT cells play key role in malignancies through their ability to rapidly produce a large amount of pro-inflammatory cytokines (Balouchi-Anaraki S et al., 2018). Indeed, smoking is also involved because it was reported to induce inflammatory responses and obstruction of airways, and to be associated with COPD (Caramori et al., 2019). Interestingly, pro-inflammatory signals could be triggered by administration of Mt-DNA in an animal model (Rodríguez-Nuevo et al., 2019). For specific cytokines, increased TNF-α and IL-10 were associated with COPD and lung cancer (Chen et al., 2020). In addition, level of IL-6 was higher in lung cancer than in COPD patients (Chen et al., 2020). However, the relationships between cf-MtDNA and cytokines and their combined involvement in COPD and NSCLC have not been well understood.

The description above clearly indicates the opportunity and need to better understand etiologies and relationships between COPD and lung cancer. To our knowledge, there has not been a report on correlations between cytokines and cf-MtDNA in blood of patients with COPD and lung cancer, especially in a follow-up study. Our investigation was focused on COPD and NSCLC by characterizing involvement of cf-MtDNA and cytokines, and their values as biomarkers for the diseases. 

## Materials and Methods

*Study subjects and sample collection*

Three groups of subjects (NSCLC patients, COPD patients and normal controls) were recruited for our investigation. Each accepted subject was required to sign our written consent form for participation and the study was approved by the Medical Ethics Committee of the Astana Medical University (Nur-Sultan city, Protocol №4).

The NSCLC patients were sequentially recruited from the Akmola Regional Oncology Center (Kokshetau city) and the Oncology Research Institute (Nur-Sultan city) during 2015 and 2019. Blood samples were collected from them before they had any therapy and they were followed-up to document their clinical courses, e.g., at different stages of the disease, metastasis and survival. The clinical stages were classified according to the eighth edition of the TNM Classification of Malignant Tumors (Brierley et al., 2017). As long as patients were able to donate blood samples, there was no particular exclusion criteria. Each accepted patient was interviewed to obtain personal information (e.g., gender, age, smoking status) and medical history (e.g., history of COPD). 

COPD patients were recruited from the Pulmonology Department of the Multidisciplinary city hospital № 2 (Nur-Sultan city) during 2018 and 2019. They were clinically validated to have the disease based on the global COLD (Global Initiative for Chronic Obstructive Lung Disease) strategy [http://goldcopd.org/wp-content/uploads/2017/11/GOLD-2018-v6.0-FINAL-revised-20-Nov_WMS.pdf]. In addition, they had no other lung diseases nor lung cancer. These patients were also interviewed as described for the lung cancer patients.

Normal subjects (controls) were recruited from the Transfusion Research Center (Nur-Sultan city). They were recruited with the general characteristics which matched the two groups of patients. The inclusion criteria were: in good health, and with health screening confirmation for absence of pulmonary pathologies and acute/chronic inflammatory diseases. There were no particular exclusion criteria. 

In the respective clinics, approximately 10 ml of blood were taken by registered nurses from each subject into BD vacutainer blood collection tubes which contained EDTA as anticoagulant. The samples were transported to the laboratory in refrigerated containers and to be analyzed using different assays.

*Determination of cf-MtDNA copy numbers in blood plasma*

Blood samples from each subject were centrifuged at 3,000 rpm for 15 min to collect plasmas. Total cf-DNA was isolated using the PROBA-NK reagent kit (#D07-2, DNA-Technology, Russia) according to the manufacturer’s protocol. The Mt-specific 16s-RNA was used as the target for detecting presence and changes in copy numbers for cf-Mt-DNA. Specific primers for a 230-bp fragment were designed and the sequence for the forward primer was: 5′-CAGCCGCTATTAAAGGTTCG-3′ and reverse primer: 5′-GGGCTCTGCCATCTTAACAA-3′. A standard curve was created according to a protocol (Ellinger et al., 2008). In the protocol, each 25µL reaction mixture contained 1X Thermo Scientific Maxima SYBR Green/ROX qPCR Master Mix (2X) (#K0222, Thermo Fisher Scientific, USA), 20 pmol forward/reverse primer and 100 ng of DNA sample. qPCR conditions were 90°C for 10 min, followed by 40 cycles at 95°C for 15 s and 60°C for 60s. All experiments were performed in duplicates on the CFX96 Touch Real-Time PCR Detection System (Bio-Rad, USA) as described above. Melting-curve analyses were conducted to confirm specificity of the PCR products. PCR products were purified by using the QIAquickR PCR Purification kit (#28106, Qiagen, Germany) according to the manufacturer’s protocol. DNA concentrations were determined using the Quanti-iTTM PicoGreenTM dsDNA Assay Kit (#P11496, Thermo Fisher Scientific, USA) according to the manufacturer’s protocol. The cf-MtDNA copy numbers were calculated according to the protocol ‘Creating Standard Curves with Genomic DNA or Plasmid DNA Templates for Use in Quantitative PCR’ (Applied Biosystems) [http://www6.appliedbiosystems.com/support/tutorials/pdf/quant_pcr.pdf].

*Determination of IL-6, and TNF-a in blood plasma*

Plasma levels of IL-6, TNF-α were measured using ELISA kits: Interleukin-6-IFA-Best (A-8768, Vector-Best, Russia) and Alpha-TNF-IFA-Best (A-8756). According to the ELISA instructions, samples were added to 96-microtiter wells which were pre-coated with the specific monoclonal antibodies from the kits and incubated at room temperature for 2 h. Then, samples with biotinylated monoclonal antibodies were incubated for 1 h at room temperature. After washing three times with the Wash Buffer from the kit, the enzyme Streptavidin-HRP (also from the ELISA kit) which binds the biotinylated antibody, was added and incubated further for 30 min. The plates were washed three more times with the Wash Buffer. Chromogen TMB (3,3’, 5,5;-tetramethylbenzidine) substrate solution was added to induce colored reaction products. The absorbance at 450 nm of each well was determined using a microplate reader (Asys Expert microplate reader; Biochrom, Ltd., Cambridge, UK). The tests for all samples were performed in triplicates. 

*Statistical analysis*

The χ-square method was used to assess statistically significant differences in gender, age, and smoking status between patients with NSCLC or COPD and the normal controls. cf-Mt-DNA copy numbers were compared using the Mann-Whitney test. One-way analysis of variance (Anova) followed by multiple comparisons with Tukey’s method were used to analyze the levels of IL-6 and TNF-α from patients and healthy individuals. Correlations between cf-Mt-DNA copy numbers and cytokines were calculated using the Spearman’s rank correlation coefficients. Survival curves were drawn using the Kaplan-Meier method. The long-rank test was used for comparisons of data. P<0.05 was considered to indicate statistically significant differences. All statistical analyses were performed using GraphPad Prism 7 software (GraphPad Software, Inc., La Jolla, CA, USA).

## Results

The comparative characteristics of patients with NSCLC and COPD, and the controls are listed in Table 1. Among these volunteers, 62 normal individuals were recruited but two were rejected because they were former smokers. There were 45 COPD and 64 NSCLC patients. As shown in the table, there was no statistical differences in the collected characteristics among the three groups of participants at the time when blood samples were collected. 

Blood samples from the 64 NSCLC patients were collected for laboratory analyses before they had received any therapy. Then, the patients were followed for a maximum of 5 years to collect clinical information. Their hospital clinical diagnosis at admission indicated that 16 of them had stage I, 19 stage II, 21 stage III and 8 stage IV of the disease. During the 5 years, 9 patients had metastasis and 25 patients had died. 

*Levels of cf-MtDNA in blood plasma of healthy individuals*

Among the 60 healthy individuals, the mean cf-MtDNA copy number was 7.23х10^5^ copies/ml. The minimum and maximum values were from 6.02×10^3^ and 1.73×10^7^ copies/ml, respectively. The copy number for males was 7.71x10^5^ copies/ml and for females was 7.23x10^5^ copies/ml. For different age groups: the copy number for <45 yo was 7.35x10^5^ copies/ml, < 55 yo was 7.6x10^5^ copies/ml, >55 yo was 4.79x10^5^ copies/ml. There was no significant effect of age and sex on the copy numbers.

*Effect of smoking on cf-MtDNA copy numbers among healthy individuals*

Among these healthy individuals, 23 were regular cigarette smokers for > five years, and 37 were non-smokers. Two volunteers were excluded from this study because they were former smokers.

From our analyses, the average cf-MtDNA level was 7.84x10^5^ copies per ml in smokers and 5.00x10^5 ^for non-smokers, the difference was significant (P = 0.001). The range of copy numbers for the smokers was 1.24x10^4^ - 1.73x10^7^, and for the non-smokers was 6.02x10^3^ - 1.12x10^6^.

*Levels of cf-MtDNA copy numbers in COPD patients*

**Figure 1:**
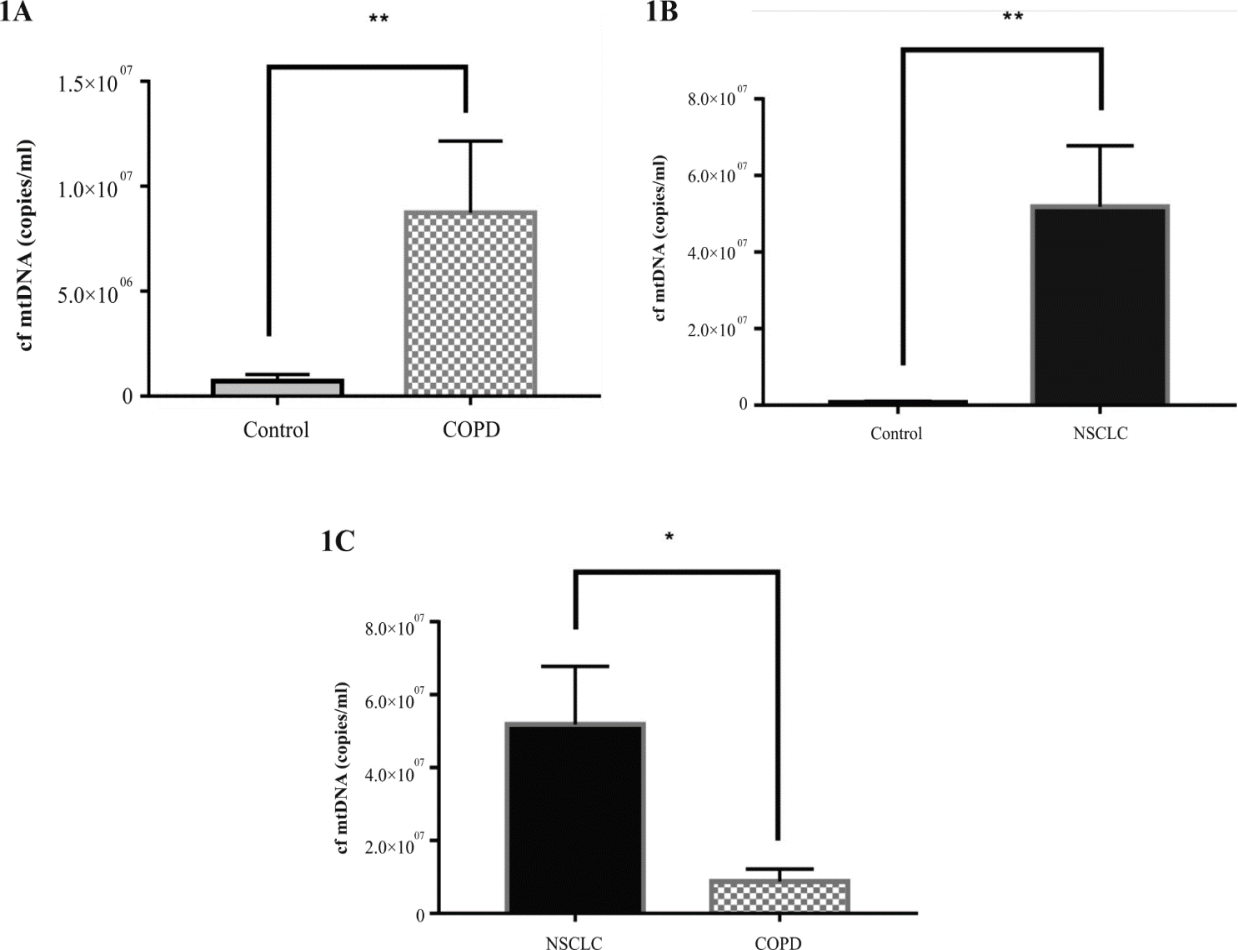
Copy Numbers of Cell-Free Mitochondrial DNA (cf-MtDNA) in Patients with Chronic Obstructive Pulmonary Disease (COPD) (1A), in Non-Small Cell Lung Cancer (NSCLC) (1B) Patients Compared to Controls and in NSCLC Patients Compared to Patients with COPD (1C)

The mean level of cf-MtDNA among the 45 COPD patients was 8.74х10^6^ copies/ml which was significantly higher than that among normal controls (P=0.0072; Figure 1A). The range was 9.31х10^4^ - 1.33х10^7^. Among the patients who were smokers, the mean level was 9.97x10^6 ^copies/ml. The copy number was 9.58x10^6^ for former smokers and was 8.74x10^6^ for non-smokers. There was no significant difference among the three groups of COPD patients, with respect to their smoking status. In addition to the clinical diagnosis for severity of COPD, the patients were also evaluated using the Medical Research Council’s Dyspnoea Scale to assess severity of shortness of breath (Hsu et al., 2013). There was no association between the cf-MtDNA copy numbers and severities for the two parameters. 

*Levels of cf-MtDNA among NSCLC patients*

The average copy number of cf-MtDNA in the 64 untreated patients was 5.18x107 copies/ml which was significantly higher than that in controls (P = 0.002; Figure 1B). Our further analyses indicate that the average copy number for the NSCLC patients was 6X and significantly higher than that in patients with COPD (P = 0.027; Figure 1C). The average copy number for smokers was higher but not significantly over the non-smokers. Furthermore, the copy numbers did not show significant differences among patients who had different stages of NSCLC. For example, the 16 stage I patients had, on average, 5.32 × 10^7^ copies per ml cf-MtDNA, 19 stage II patients had 5.52 × 10^7^, 21 stage III patients had 4.04 × 10^7^ and 8 stage IV patients had 3.95 × 10^6^. However, for the 9 patients who subsequently developed metastases, the average copy number was 8.22x10^7^ which was significantly higher than that in the 55 patients who had no metastases: 5.18x10^7^, P = 0.0001. 

*Survival analysis of NSCLC patients according to cf-MtDNA levels*

**Figure 2:**
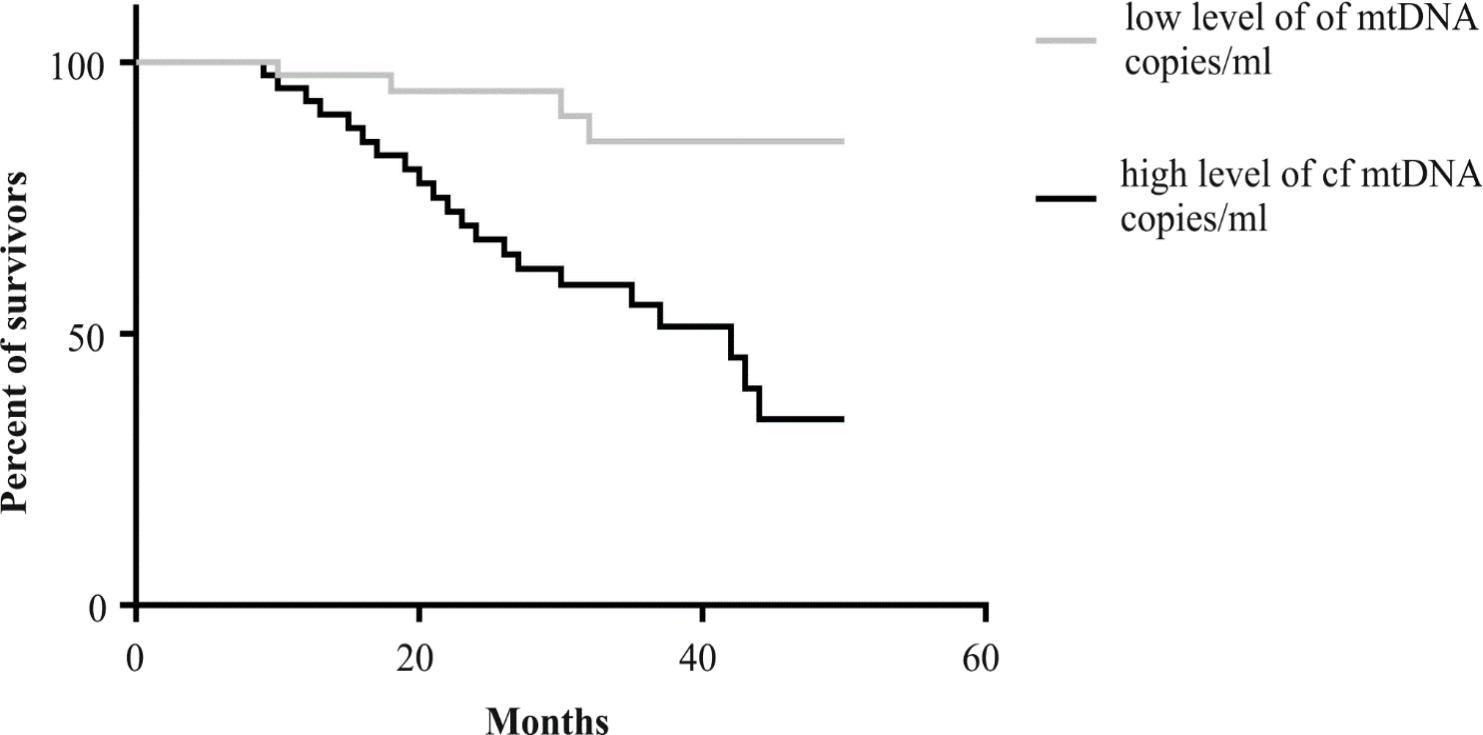
Kaplan-Meier Survival Curves for NSCLC Patients with Different Levels of cf-MtDNA

The 64 NSCLC patients were followed-up by doctors for a maximum of 5 years from the time of diagnosis. From the collected information, the median survival values were used for association analyses with the previously determined high and low levels of cf-MtDNA. The calculated relative risk (HR = 3.9; 95% CI 1.78-8.58, P<0.01) indicates that having high copy numbers of cf-MtDNA was predictive of poor prognosis among the patients (Figure 2).

*Levels of IL-6 in patients with NSCLC and COPD patients, and in controls*

**Figure 3:**
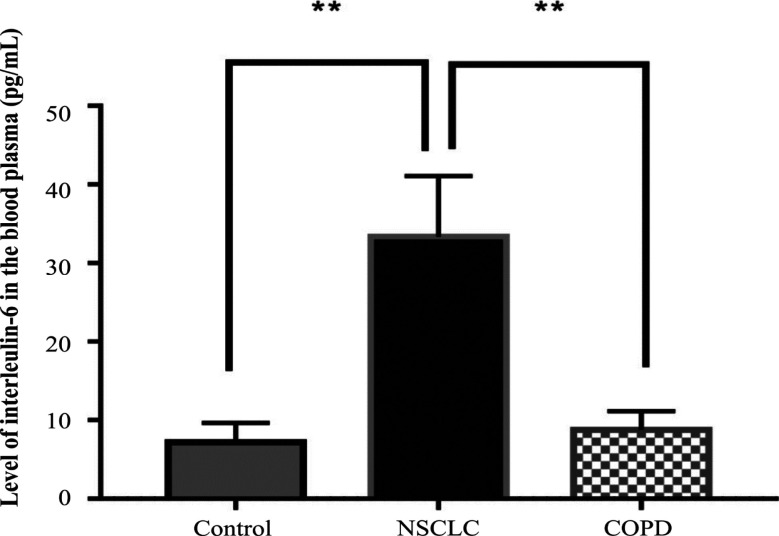
Levels of IL-6 in NSCLC and COPD Patients, and Controls

The average levels of IL-6 in the untreated NSCLC patients, COPD patients and controls were 33.4±7.7 pg/ml, 8.8±2.4 pg/ml and 7.2±2.4 pg/ml, respectively. The level in the NSCLC patients was significantly higher than that of the other two groups (P < 0.009; Figure 3).

*Levels of TNF-α in NSCLC and COPD patients, and in controls*

**Figure 4:**
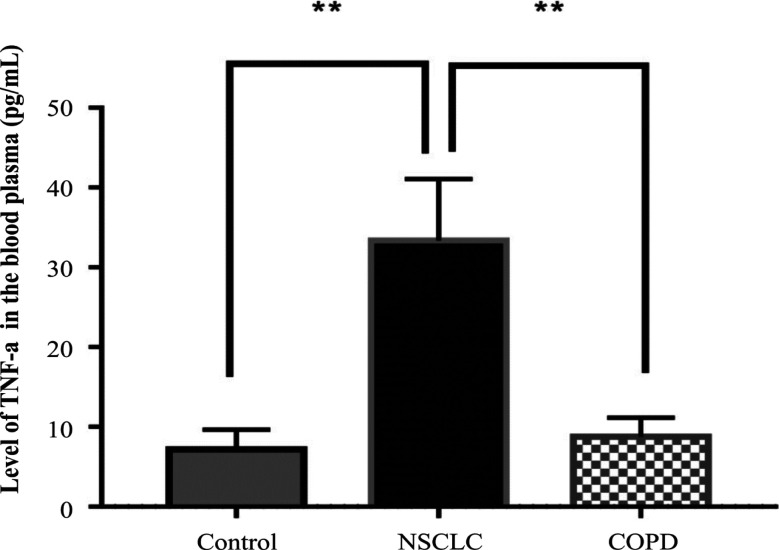
Levels of TNF-a in NSCLC and COPD Patients, and Controls

The average levels of TNF-α in the NSCLC and COPD patients, and controls were 10.0 ± 0.5 pg/ml, 6.5 ± 0.5 pg/ml and 5.8 ± 1.0 pg/ml, respectively. The levels in the NSCLC patients were significantly higher than that in the other two groups (P < 0.001; Figure 4).

*Correlation of proinflammatory cytokine levels and cf-MtDNA copy numbers in NSCLC patients*

**Figure 5:**
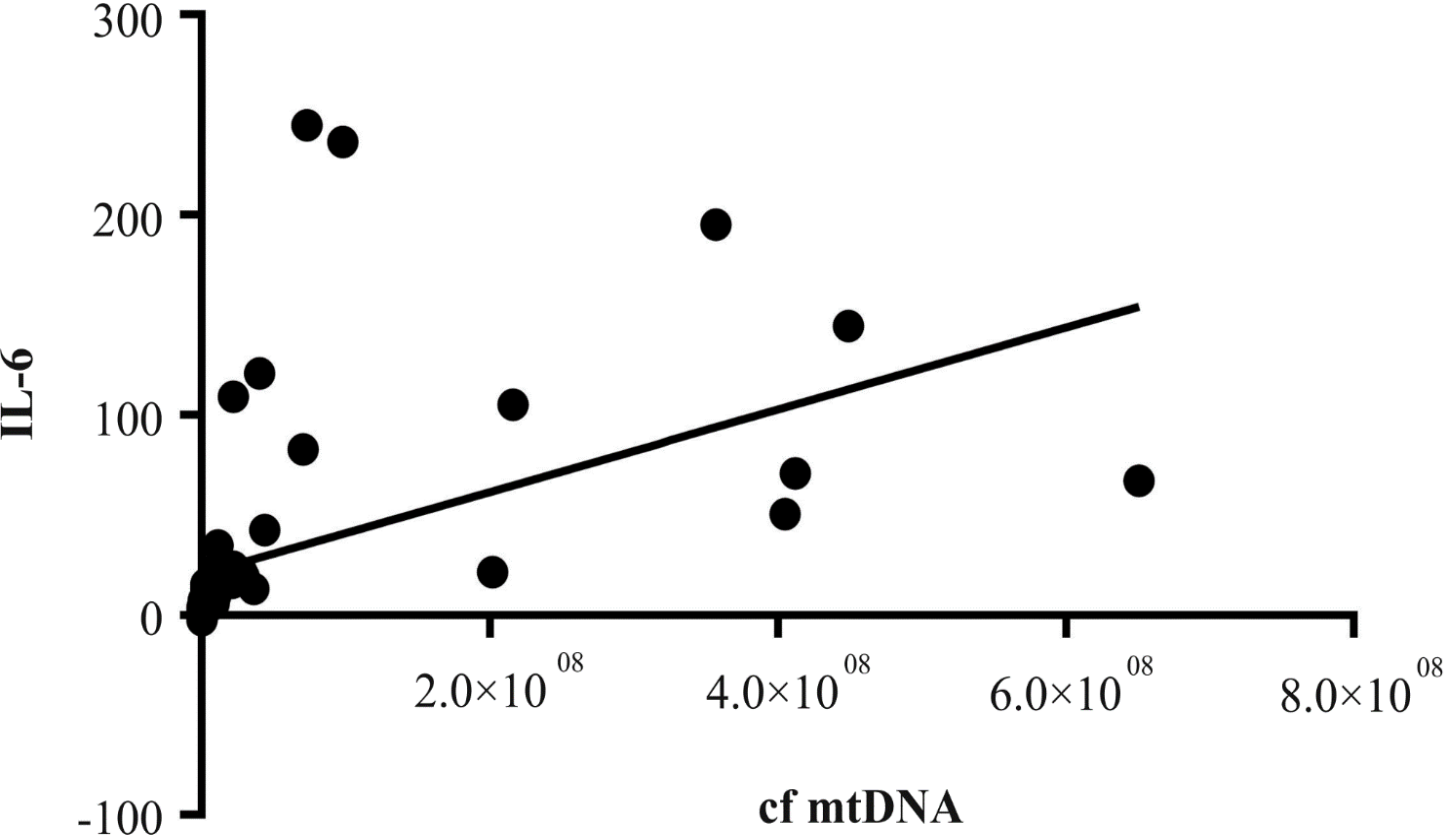
Correlation of IL-6 and cf-MtDNA Copy Numbers in NSCLC Patients

Our data indicate that IL-6 expression was significantly and positively correlated with cf-MtDNA copy numbers (r= 0.9; P<0.001; CI95% 0.87 -0.95; Figure 5) but not for TNF-α (r= 0.07; P= 0.58; CI95% 0.04 -0.19).

**Table 1:** Characteristics of the NSCLC and COPD Patients in Comparison with the Controls*

	Control	NSCLC	P value	COPD	P value
Male	36	48	Р=0.94	32	Р=0.29
Female	26	16		13	
Age ≤60	58	21	Р=0.99	12	Р=0.86
Age>60	4	43		33	
Non-smokers	37	25	Р=0.91	20	Р=0.62
Smokers	23	39		13	
Former smokers	2	0		12	

*NSCLC and COPD, non-small cell lung cancer and chronic obstructive lung disease patients

## Discussion

The involvement of smoking in release of mtDNA is further clarified from our investigation. Our data show that among healthy controls, cf-MtDNA copy numbers were significantly higher among smokers than non-smokers. The copy numbers were non-significantly higher among COPD patients than controls. For NSCLC patients, the average copy number was significantly higher, whether they were smokers or not, than that in the controls. Furthermore, these NSCLC patients had the highest copy numbers among the three groups (p < 0.002). Therefore, smoking played an important role for development of the two lung diseases (as well-known already) but the role may be mediated via cell/tissue injury in the lung which released cf-MtDNA and activated proinflammatory cytokines into body fluid. This observation also suggests that cf-MtDNA can also be used as an effective biomarker to provide early warning signal for the two lung diseases. 

Besides smoking, other factors which cause cell/tissue injuries can also lead to the release of cf-MtDNA. For example, apoptosis is one of the main sources of cf-MtDNA (Thurairajah et al., 2018). In addition, it has been demonstrated that metastatic breast cancer cells can induce neutrophil extracellular traps (NETs) production in the absence of infectious agents (Park et al., 2016). NETs can also be sources of cf-MtDNA (Xu et al., 2015; Thurairajah et al., 2018). cf-MtDNA can circulate in the blood as a result of necrosis too (Thurairajah et al., 2018), including necrosis of tumor cells (Agostini et al., 2011). 

Increase of cf-MtDNA has been associated with COPD and, with some contradictory reports, lung cancer (Chen et al., 2018; Hou et al., 2019; Zhang et al., 2020). Results from our study show that the levels of cf-MtDNA in patients with COPD and NSCLC were significantly higher than that in the controls. 

More importantly, the increases in copy numbers which were detected before the NSCLC patients had received therapy was predictive of unfavorable outcomes of these patients, i.e., their subsequent metastasis and mortality within 5 years. Therefore, with additional validation, the cf-MtDNA can also be a useful biomarker to monitor prognosis of NSCLC patients.

The release of cf-MtDNA into body fluids can have other biological consequences. For example, increase of cf-MtDNA which was caused by injury in a rat model led to the development of inflammatory processes in the lungs (Gan et al., 2015). Regarding inflammation, results from our study show a significant increase in the levels of proinflammatory cytokines, interleukin-6 and TNF-a, in the NSCLC patients, not only compared to the controls, but also to patients with COPD. The role of cf-MtDNA as a key influence on inflammation was first reported by Collins and colleagues (Collins et al., 2014), who demonstrated that intra-articular injections of mtDNA, but not nuclear DNA, contributed to the development of arthritis in mice. Lung tissues were characterized to have higher susceptibility to cf-MtDNA exposure compared to other tissues (Zhang et al., 2014). Currently, there are in vivo studies using exogenous mtDNA to provoke local or systemic inflammation. For example, it was shown that intratracheal administration of mtDNA in rats provoked the development of pneumonia via the TLR9-p38 MAPK signaling pathway (Gu et al., 2015). Chronic inflammation was reported to promote tumor progression and to support spreading of metastasis (Multhoff et al., 2012; Comen et al., 2018). However, the role of cf-MtDNA in the inflammatory process has still not been well-characterized. From our investigation, the cf-MtDNA copy numbers were significantly associated with the levels of proinflammatory cytokines, especially IL-6 in the NSCLC patients. However, further studies are needed to clarify the association with TNF-α.

In summary, our investigation shows that cigarette smoking was significantly associated with increase of cf-MtDNA copy numbers in body fluid, possibly via injury to lung cells and tissues. Increased cf-MtDNA copy numbers was significantly associated with COPD and NSCLC. More importantly, the increase was predictive of adverse outcomes of NSCLC patients, particularly their subsequent development of metastasis and mortality in less than 5 years. Our data also indicate that increased cf-MtDNA copy numbers was significantly associated with expression of inflammatory cytokines, especially IL-6, which served to aggravate pathogenesis of the two lung diseases. Therefore, our data show that cf-MtDNA copy numbers were indicative of lung cell/tissue injuries, of release of pro-inflammatory cytokines, of development of the two lung diseases, and of poor prognosis for NSCLC. Consequently, cf-MtDNA can be considered for use as a biomarker to provide early warning signals for the two lung diseases and for prognosis determination of NSCLC.

## Author Contribution Statement

(I) Conception and design: R. Bersimbaev. (II) Administrative support: R. Bersimbaev. (III) Provision of patients: N. Kalibekov, D. Serikbaiuly. (IV) Collection and assembly of data: A. Kausbekova, A. Kussainova. (V) Data analysis and interpretation: O. Bulgakova. (VI) Manuscript writing: All authors. (VII) Final approval of manuscript: All authors.

## Data Availability

The analyzed data sets generated during the study are available from the corresponding author on reasonable request.
